# Fire Retardancy and Dielectric Strength of Cyclotriphosphazene Compounds with Schiff Base and Ester Linking Units Attached to the Electron-Withdrawing Side Arm

**DOI:** 10.3390/polym14204378

**Published:** 2022-10-17

**Authors:** Siti Nur Khalidah Usri, Zuhair Jamain, Mohamad Zul Hilmey Makmud

**Affiliations:** 1 Organic Synthesis and Advanced Materials (OSAM) Research Group, Faculty of Science and Natural Resources, Universiti Malaysia Sabah, Kota Kinabalu 88400, Sabah, Malaysia; 2 Future Material and Sustainable Energy (FuSE) Research Group, Faculty of Science and Natural Resources, Universiti Malaysia Sabah, Kota Kinabalu 88400, Sabah, Malaysia

**Keywords:** cyclotriphosphazene, electron withdrawing, fire retardant, dielectric, side arm

## Abstract

A series of compounds with Schiff base and ester linking units attached to the electron-withdrawing side arm (Cl, NO_2_, and OH) have been successfully synthesized through four schemes of the chemical route. These compounds were characterized using Fourier Transform Infrared spectroscopy (FTIR), Nuclear Magnetic Resonance spectroscopy (NMR), and Carbon, Hydrogen and Nitrogen (CHN) elemental analysis. The epoxy resin was used as a matrix of molding to observe the refinement of fire-retardant properties of the modified cyclotriphosphazene compounds. The fire-retardant testing was done using Limiting Oxygen Index (LOI). The LOI value of pure epoxy resin was increased from 22.75% to 24.71% when incorporated with 1 wt.% of hexasubstituted cyclotriphosphazene (HCCP). Interestingly, all the final compounds gave a positive increment in the LOI value and the highest LOI value was obtained from the compound containing a nitro side arm with LOI value of 26.90%. In order to understand the thermal stability of these compounds, Thermogravimetric Analysis (TGA) was carried out. The compound with the nitro group at the terminal end has the highest char residue which is 34.2% at 700 °C. This indicated that the presence of the nitro withdrawing group was able to enhance the fire retardancy of the materials. Based on SEM observation, the shape of the final compound’s char residue demonstrated the formation of a porous protective layer with a dense surface. The dielectric property was conducted according to ASTM D149 AC breakdown voltage to determine its dielectric strength. The results showed that the highest dielectric strength value belonged to the compound containing a nitro group side arm with 24.41 kV/mm^−1^ due to the π electron delocalization.

## 1. Introduction

Polymer and polymer composites have unique properties such as excellent fire retardant, dielectric, and mechanical strength [1,2,3]. They have numerous applications such as electrical, automobile manufacturing, and chemical industries [4,5,6,7]. Nowadays, epoxy resin has attracted much attention from researchers to enhance and improve its properties. Due to its excellent properties and outstanding performance, epoxy resins are appealing for a number of applications, for instance, composites, electrical insulation, and adhesion [8]. However, since epoxy resin is flammable in property, these will significantly restrict its ability to be used in areas that require a great need for fire resistance. In addition, Chang et al., 2021 also reported that epoxy polymer-based dielectric materials play a crucial role in the advancement of electric devices since it requires a complex equipment design with a high voltage stress which imposes stringent requirements, such as a high dielectric strength on epoxy materials [9]. In the case of application materials, especially epoxy resin in electrical and electronic systems, the combination of these two properties is important to study in order to overcome the major issue related to these two properties in epoxy resin. Therefore, many researchers have attempted to lessen the fire risk and enhance the performance of the dielectric strength property in epoxy resin.

Generally, fire-retardant epoxy resin is typically created by a combination of a halogenated compound with epoxy resin. However, halogenated-based flame retardants are reported to be harmful to the environment as they release toxic and corrosive substances during their decomposition, leading to environmental problems and threatening human health [10,11,12]. Recently, in consideration of the environmental problems, Gao et al. (2021) have reported that the luteolin-based epoxy resin work efficiently in inhibiting the release of flammable volatile gases and reducing toxic and hazardous properties. This research also revealed that the fire retardant and heat resistant epoxy resin of this compound have a great potential for industrial and domestic applications [13]. In addition, to address this issue, non-halogenated flame retardance has also been thoroughly studied and applied [14,15,16]. Based on the previous study, phosphorus- and nitrogen-containing molecules are outstanding compounds that have been demonstrated as excellent fire-retardant materials [17,18]. As a result, the modification of non-halogenated derivatives showed a rapidly growing interest in the research field related to fire-retardant and dielectric studies [19,20].

Cyclotriphosphazene derivatives are regarded as important P/N flame retardants due to their facile molecular design, excellent thermal stability, and outstanding fire-retardant and dielectric properties [21,22,23,24]. Zarybnick and his co-worker have reported that the cyclotriphosphazene-based curing agent had an effect on the flame resistance of epoxy resin [25]. This study synthesized two compounds, HCACTP and DTCATP, and were applied as curing agents for epoxy resin. The study was aimed to investigate the fire retardancy performance of the synthesized compounds and how they acted as curing agents. The results revealed that the application of the derivatives gave a the facelift to fire retardancy performance of the epoxy resin. In addition, Jamain et al. (2020) also studied fire retardancy properties of polyester resin with hexasubstituted cyclotriphosphazene compounds containing Schiff base and amide linking units [26]. This study discloses that the value of the LOI of polyester resin increased when it was incorporated with a hexasubstituted cyclotriphosphazene compound containing Schiff base and amide linking units. The value of the LOI increased from 22.53% to 24.71%.

Many studies have reported on the excellent properties of hexasubstituted cyclotriphosphazene in enhancing the fire-retardant properties in polymer, such as epoxy resin and polyester resin [27,28,29,30]. However, the study of new hexasubstituted cyclotriphopsphazene compounds containing Schiff base and ester linking units attached to the electron withdrawing side arm has not been reported. Moreover, the study on the structure-property relationship was also conducted to correlate the structure’s effect, which can contribute to enhancing the property of epoxy resin towards its fire-retardant behavior. In addition, this study also aims to observe dielectric properties, especially dielectric strength, since epoxy resin was claimed to have excellent dielectric properties and has been used in many electrical applications such as encapsulating electrical components [31,32].

Hence, it is important to prepare new cyclotriphosphazene derivatives to improve epoxy resin’s fire-retardant and dielectric properties without altering its other properties. In this work, hexasubstituted cyclotriphosphazene containing the electron-withdrawing side arm (Cl, NO_2_, and OH) was synthesized throughout the condensation and esterification reaction. The chemical structure of the synthesized compounds was characterized using Fourier Transform Infrared (FTIR), Nuclear Magnetic Resonance (NMR), and CHN elemental analysis. The flame retardancy and dielectric properties of the compound, which is incorporated with epoxy resin (EP), have been studied in detail using LOI test and alternating current (AC) breakdown voltage. TGA and SEM were conducted in order to study the thermal stability and char effect toward fire retardancy.

## 2. Materials and Methods

All the intermediates and compounds undergo spectroscopic chemical analysis. All the data have been analyzed and recorded in the compact data form, as illustrated in Section 2.4. This study incorporated 1 wt.% of final compounds with epoxy resin. The sample was prepared in molding and cured using DDM as a catalyst for 24 h at 50 °C.

### 2.1. Materials

The chemicals and solvents used in this work are 4-aminophenol, 4-chlorobenzaldhehyde, 4-nitrobenzaldehyde, 4-hydroxybenzaldehyde, 4-hydroxybenzoate, phosphonitrilic chloride trimer, potassium carbonate, sodium hydroxide, ethanol, dicyclohexylcarbodiimide, dimethylaminopyridine, acetone, dichloromethane, hydrochloric acid, and methanol. None of the solvents were further purified and they were utilized in their original state. All chemicals and solvents were purchased from Sigma-Aldrich (Steinheim, Germany), Acros Organics (Geel, Belgium), Merck (Darmstadt, Germany), and Qrëc (Asia) (Selangor, Malaysia).

### 2.2. Analysis and Testing Instrument

#### 2.2.1. Fourier Transform Infra-Red (FTIR)

The functional groups of the synthesis compound can be identified using FTIR spectroscopy. The infrared frequency was expressed as a wave number and was scanned in the range of 600–4000 cm^−1^. A Perkin Elmer FT-IR Microscopy Spotlight 200 (PerkinElmer, Waltham, MA, USA) was used to get the IR spectra for all intermediates and the final compound.

#### 2.2.2. Nuclear Magnetic Resonance (NMR)

For some atomic nuclei, such as H, C, and P, NMR spectroscopy is used to ascertain the molecular structure of compounds. To confirm the structure of every synthesized compound, 1D NMR is used. In this study, the NMR analysis was conducted using a Bruker 500 MHz Ultrashield spectrometer (Bruker, Coventry, UK) to collect all NMR spectra. About 20 mg of samples are dissolved in a deuterated solvent before being transferred into an NMR tube. 

#### 2.2.3. CHN Elemental Analysis

In determining the purity for all synthesized compounds, CHN elemental analysis was used in order to reveal the percentage of carbon (C), hydrogen (H) and nitrogen (N). This technique entails the burning of samples over an excess supply of oxygen. This analysis also provides a rapid and inexpensive method in determining the purity of the analyzed compounds. The accuracy of the instrument is ±3%. In this study, the analysis was carried out using CHN analyzer model Perkin Elmer II, 2400 (PerkinElmer, Waltham, MA, USA). 

#### 2.2.4. Thermogravimetric Analysis (TGA)

TGA was carried out on a thermogravimetric analyzer Q50 (PerkinElmer, Waltham, MA, USA). Specimens of about 0.1 g were heated from room temperature to 700 °C at a rate 10 °C min^−1^ in an N_2_ atmosphere.

#### 2.2.5. Limiting Oxygen Index (LOI)

A Limiting Oxygen Index (LOI) test (S.S. Instrument Pvt. Ltd., Delhi, India) is used to determine the minimum amount of oxygen needed to support the combustion of a sample. The LOI testing was performed using an FTT oxygen index, according to BS 2782: Part 1: Method 141 and ISO 4589. All the experiment was replicated three times for all compounds. 

#### 2.2.6. Scanning Electron Microscope (SEM)

SEM (Hitachi, Krefeld, Germany) was used to investigate the relationship between the morphology of the char layers and fire retardant properties of final compounds blended in PE.

#### 2.2.7. Alternating Current Breakdown Voltage 

As the standard testing for solid electrical insulating materials, ASTM D149 was used for the dielectric testing for this study (Metrohm Autolab B.V., Utrecht, The Netherlands). ASTM D149 tested the dielectric strength between 25 and 800 Hz. This test determines the breakdown voltage through the thickness of the test materials. The sample was placed in the chamber that contains the anode and cathode, and the electrode of the sample was connected to a circuit to observe the alternating current (ac) breakdown voltage. The sample was immersed in silicon oil with a voltage application of 60 Hz to the anode electrode, while the cathode electrode was grounded. The voltage was monitored from the start of the voltage application until the breakdown occurred. 

### 2.3. Preparation of Samples

For fire retardant and dielectric properties testing, two types of samples were prepared according to the standard dimension for both testing. For fire retardant testing, 1 wt.% of the final compound was mixed with epoxy resin. The mixture of the final compound with epoxy resin was cured with DDM with 1:1 molar ratio. The mixture was stirred constantly at room temperature to ensure the mixture is homogenous. The mixture was then poured into the mold with a given dimension for fire retardant testing which was 120 mm × 10 mm × 4 mm and placed in the oven for 24 h at 50 °C. For dielectric testing, the same sample method was used for preparing the sample, but the dimension used was 2 mm thickness and 2 cm diameter in a round shape mold. 

### 2.4. Synthesis Methods 

**Scheme 1:**
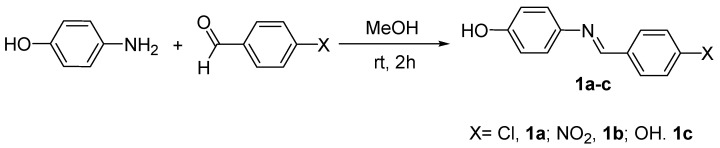
Synthesis pathway of intermediates **1a**–**c**.

**Scheme 2:**
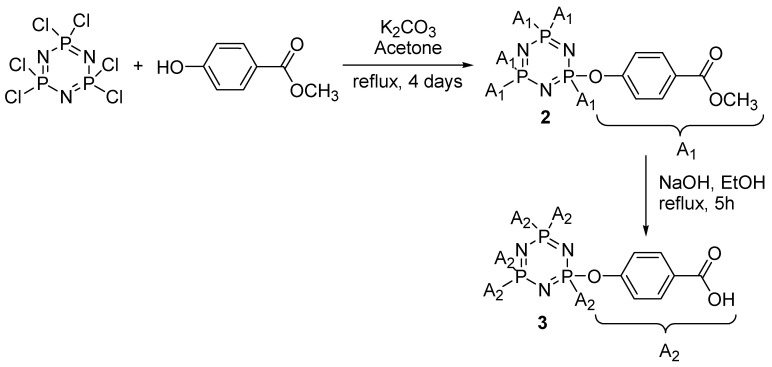
Synthesis pathway of intermediates **2** and **3**.

**Scheme 3:**
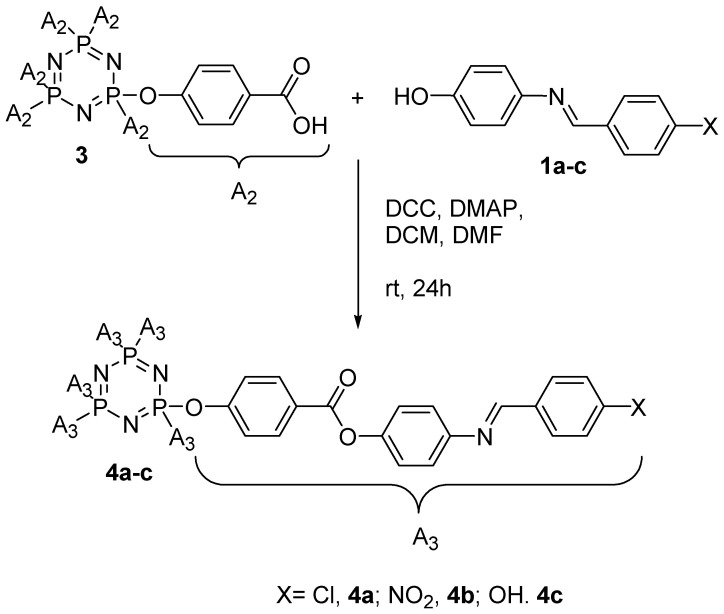
Synthesis pathway of hexasubstituted cyclotriphosphazene compounds, **4a**–**c**.

Condensation reaction of 4-substitutedbenzaldehydes (Cl, NO_2_, and OH) with 4-aminophenol yielded intermediates **1a**–**c** (Scheme 1) [33]. Another reaction of methyl-4-hydroxybenzoate with phosphonitrilic chloride trimer produced intermediate **2**, which further reduced to form intermediate **3** with carboxyl terminal group (Scheme 2) [34,35]. The esterification of **3** and **1a**–**c** formed new hexasubstituted cyclotriphosphazene compounds, **4a**–**c** (Scheme 3) [36].

#### 2.4.1. Synthesis of 4-[(4-substitutedbenzylidene)amino]phenol, **1a**–**c**

A solution of 4-aminophenol (0.02 mol) and 4-chlorobenzaldehyde (0.02 mol) in 40 mL of methanol was mixed in 250 mL round bottom flask. The mixture was stirred for 2 h at room temperature. The reaction progress was monitored using TLC. Upon completion, the precipitate form was filtered, washed with cold water and dried overnight. The mixture was recrystallized using methanol. The same method was used to synthesize **1b**–**c**.

4-[(4-chlorobenzylidene)amino]phenol, **1a**

Yield: 4.271 g (92.16%), Orange powder. FTIR (cm^−1^): 3086 (OH stretching), 1505 (C = C stretching), 1618 (C = N stretching), 1273 (C-O stretching), 1273 (C-N), 768 (C-Cl stretching). ^1^H-NMR (500 MHz, DMSO-d_6_), δ, ppm: 9.57 (s,1H), 8.61 (s,1H), 7.91 (d,2H), 7.55 (d,2H), 7.22 (d,2H), 6.82 (d,2H). ^13^C-NMR (125 MHz, DMSO-d_6_), ppm: 156.97, 156.24, 142.69, 135.82, 130.29, 129.32, 123.09, 116.21, 40.45. CHN elemental analysis calculated for C_13_H_10_ClNO: C: 67.40%, H: 4.35%, N: 6.05%; Found: C: 67.14%, H: 4.25%, N: 5.94%.

4-[(4-nitrobenzylidene)amino]phenol, **1b**

Yield: 4.271 g (92.16%), Brown powder. FTIR (cm^−1^): 3429 (OH stretching), 1505 (C = C stretching), 1623 (C = N stretching), 1262 (C-O stretching), 1162 (C-N). ^1^H-NMR (500 MHz, DMSO-d_6_), δ, ppm: 8.77 (s,1H), 8.32 (d,2H), 8.13 (d,2H), 7.31 (d,2H), 6.84 (d,2H). ^13^C-NMR (125 MHz, DMSO-d_6_), ppm: 157.68, 155.22, 142.69, 135.82, 130.29, 129.32, 123.09, 116.21, 40.45. CHN elemental analysis calculated for C_13_H_10_N_2_O_3_: C: 64.46%, H: 4.16%, N: 11.56%; Found: C: 64.01%, H: 4.11%, N: 11.47%.

4-[(4-hydroxybenzylidene)amino]phenol, **1c**

Yield: 4.125 g (96.81%), Brown powder. FTIR (cm^−1^): 3525 (OH stretching), 1506 (C = C stretching), 1589 (C = N stretching), 1235 (C-O stretching), 1165 (C-N). ^1^H-NMR (500 MHz, DMSO-d_6_), δ, ppm: 8.69 (s,1H), 8.04 (s,1H), 8.00 (d,2H), 7.26 (d,2H), 6.82 (d,2H). ^13^C-NMR (125 MHz, DMSO-d_6_), ppm: 167.47, 157.19, 142.58, 140.59, 132.87, 130.15, 128.70, 123.27, 116.07, 40.32. CHN elemental analysis calculated for C_13_H_11_NO_2_: C: 73.23%, H: 5.20%, N: 6.57%; Found: C: 73.01%, H: 5.11%, N: 6.48%.

#### 2.4.2. Synthesis of Hexakis(4-Benzoate-Phenoxy)Cyclotriphosphazene, **2**

In a 250 mL round bottom flask, a mixture of methyl 4-hydroxybenzoate (0.07 mol), phosphonitrilic chloride trimer (0.01 mol) and potassium carbonate, K_2_CO_3_ (0.1 mol) was mixed with acetone (150 mL). The mixture was refluxed for 4 days. The reaction progress was monitored using TLC. Upon completion, the mixture was poured into cool water (250 mL). The precipitate formed was filtered, washed with water, and dried overnight to give a white solid. 

Yield: 10.01 g (79.41%), White powder. FTIR (cm^−1^): 1602 (C = O stretching), 1503 (C = C stretching), 1271 (C-O stretching), 1161 (P = N), 956 (P-O-C stretching). ^1^H-NMR (500 MHz, DMSO-d_6_), δ, ppm: 7.79 (d,2H), 7.55 (d,2H), 7.05 (d,2H), 3.87 (m,3H). ^13^C-NMR (125 MHz, DMSO-d_6_), ppm: 165.13, 152.85, 131.08, 126.91, 120.62, 52.23, 40.09. ^31^P-NMR (500 MHz, DMSO-d_6_), δ, ppm: 7.99 (s,1P). CHN elemental analysis calculated for C_48_H_42_N_3_O_18_P_3_: C: 55.34%, H: 4.06%, N: 4.03%; Found: C: 55.28%, H: 4.01%, N: 3.98%.

#### 2.4.3. Synthesis of Hexakis(4-Carboxy-Phenoxy)Cyclotriphosphazene, **3**

Intermediate **2** (8.17 mmol) and sodium hydroxide, NaOH (0.12 mol) in ethanol (150 mL) were mixed in a 250 mL round bottom flask. The mixture was refluxed for 5 h. The reaction progress was monitored by TLC. Upon completion, the mixture was poured into cool water (250 mL). An observed clear solution was acidified with HCl until the precipitate was formed. The precipitate was filtered, washed with water, and dried to give a white solid.

Yield: 7.741 g (98.87%), White powder. FTIR (cm^−1^): 1637 (C=O stretching), 1595 (C=C stretching), 1270 (C-O stretching), 1151 (P = N), 945 (P-O-C stretching). ^1^H-NMR (500 MHz, DMSO-d_6_), δ, ppm: 7.84 (d,2H), 7.55 (d,2H), 7.00 (d,2H). ^13^C-NMR (125 MHz, DMSO-d_6_), ppm: 165.13, 152.85, 131.08, 126.91, 120.62, 52.23, 40.09. ^31^P-NMR (500 MHz, DMSO-d_6_), δ, ppm: 8.04 (s,1P). CHN elemental analysis calculated fo C_42_H_30_N_3_O_18_P_3_: C: 52.68%, H: 3.16%, N: 4.39%; Found: C: 52.54%, H: 3.08%, N: 4.31%.

#### 2.4.4. Synthesis of Hexakis[4-{((*E*)-4-(substituted)benzylidene)amino}phenyl]benzoate triazaphosphazene, **4a**–**c**

A solution of intermediate **3** (1.05 mmol) in 5 mL of DMF was mixed with a solution containing intermediate **1a** (7.31 mmol), dicyclohexylcarbodiimide, DCC (7.31 mmol), and dimethylaminopyridine, DMAP (7.31 mmol) in 30 mL of DCM was stirred at 0 °C for 1 h and another 24 h at room temperature in a 100 mL round bottom flask. A cloudy white solid was observed during the stirring process. The reaction progress was monitored by TLC. Upon completion, the precipitate was filtered, and the filtrate was collected. The filtrate was evaporated, dried, and recrystallized from methanol to give a yellow solid. The same method was used to synthesize **4b**–**c**.

Hexakis[4-{((*E*)-4-(chloro)benzylidene)amino}phenyl]benzoate triazaphosphazene, **4a**

Yield: 2.107 g (89.62%), Brown powder. FTIR (cm^−1^): 1619 (C = N stretching), 1566 (C = C stretching), 1720 (C = O stretching), 1186 (P = N stretching), 1078 (C-N), 1186 (P-O-C stretching). 1234 (C-O stretching), 825 (C-Cl stretching). ^1^H-NMR (500 MHz, DMSO-d_6_), δ, ppm: 8.70 (s,1H), 8.25 (d,2H), 8.06 (d,2H), 7.91 (d,2H), 7.49 (d,2H),7,28 (d,2H), 6.84 (d,2H). ^13^C NMR (125 MHz, DMSO-d_6_), ppm: 166.97, 157.72, 154.95, 148.72, 142.51, 138.25, 131.54, 131.10, 124.29, 116.30. ^31^P-NMR (500 MHz, DMSO-d_6_), δ, ppm: 9.48 (s,1P). CHN elemental analysis calculated for C_119_H_78_C_l6_N_9_O_18_P_3_: C: 64.36%, H: 3.51%, N: 5.63%; Found: C: 64.27%, H: 3.48%, N: 5.58%

Hexakis[4-{((*E*)-4-(nitro)benzylidene)amino}phenyl]benzoate triazaphosphazene, **4b**

Yield: 2.117 g (88.20%), Brown powder. FTIR (cm^−1^): 1602 (C = N stretching), 1508 (C = C stretching), 1733 (C = O stretching), 1181 (P = N stretching), 1078 (C-N), 1843 (P-O-C stretching). ^1^H-NMR (500 MHz, DMSO-d6), δ, ppm: 8.64 (s,1H), 8.21 (d,2H), 8.09 (d,2H), 7.91 (d,2H), 8.02 (d,2H),7,24 (d,2H), 6.81 (d,2H). ^13^C-NMR (125 MHz, DMSO-d_6_), ppm: 166.26, 157.69, 154.84, 150.27, 148.68, 142.46, 136.90 131.01, 129.29 124.21, 116.26. ^31^P-NMR (500 MHz, DMSO-d_6_), δ, ppm: 9.54 (s,1P). CHN elemental analysis calculated for C_119_H_78_N_15_O_30_P_3_: C: 62.59%, H: 3.41%, N: 9.12%; Found: C: 62.50%, H: 3.35%, N: 9.06%

Hexakis[4-{((*E*)-4-(hydroxy)benzylidene)amino}phenyl]benzoate triazaphosphazene, **4c**

Yield: 1.977 g (88.53%), Brown powder. FTIR (cm^−1^): 1601 (C = N stretching), 1509 (C = C stretching), 1752 (C = O stretching), 1156 (P = N stretching), 1186 (C-N), 1003 (P-O-C stretching). ^1^H-NMR (500 MHz, DMSO-d_6_), δ, ppm: 8.42 (s,1H), 7.96 (d,2H), 7.74 (d,2H), 7.13 (d,2H), 6.89 (d,2H), 6.81 (d,2H), 6.63 (d,2H). ^13^C NMR (125 MHz, DMSO-d_6_), ppm: 167.47, 157.19, 142,58, 140.58, 132.87, 130.15, 128.70, 123.27, 116.07,40.32. ^31^P-NMR (500 MHz, DMSO-d_6_), δ, ppm: 9.58 (s,1P). CHN elemental analysis calculated for C_119_H_84_O_24_P_3_: C: 67.70%, H: 3.98%, N: 5.92%; Found: C: 67.53%, H: 3.88%, N: 5.87%

## 3. Results and Discussion

### 3.1. CHN Elemental Analysis

The final compound was characterized using a CHN elemental analysis to determine the purity of the compound. Table 1 shows the CHN elemental analysis data that has been performed on all final compounds.

The experimental percentage values of C, H, and N show excellent correlation with the theoretical percentage values, as shown in Table 1. There are below 3% percentage errors found between the experimental and theoretical values, which correspond to the high purity of the synthesized compound. If the results are inconsistent and have significant error rates, it may be because a bigger sample size was utilized for the analysis, which led to a lack of oxygen needed for the combustion to complete. If too many ashes are produced, there will be less nitrogen overall. The analyzed samples, especially the organic samples, have a significant impact on the CHN analyzer’s accuracy and precision of results. Poor sample preparation, in particular, may lead to sample mistakes and solvent or moisture residue production following any action with the analyzed sample, while volatility may cause changes to the variation within the composition. All of the aforementioned factors could skew the findings of the analyzed elemental solvents [37].

### 3.2. FTIR Spectral Discussion

The reaction of 4-aminophenol with 4-(substituted)benzaldehyde produced the Schiff base intermediates, **1a**–**c**. This compound showed a broad adsorption of the O-H stretching in the region of 3220–3550 cm^−1.^ The adsorption bands for C = N, C = C, and C-N were absorbed at 1617, 1535, and 1159 cm^−1^, respectively. Absorption at 790 cm^−1^ was assigned for C-Cl stretching of intermediate **1a**. There was no absorption band of the aldehydic C-H stretching in all intermediates, but instead all intermediates showed the C = N stretching at 1617 cm^−1^, which confirmed the Schiff base moiety confirmation.

The intermediate **2** has been synthesis by a substitution reaction of methyl-4-hydroxybenzote with cyclotriphosphazene (HCCP). The FTIR spectrum of intermediate **2** showed the absorption 1602, 1503, and 1271 cm^−1^ for the C = O, C = C, and C-O stretching, respectively. A strong absorption at 1161 cm^−1^ was attributed for P-N stretching, while the absorption at the region of 956 cm^−1^ was assigned to P-O-C bending. The intermediate **2** then undergo a reduction reaction to form intermediate **3**. The IR spectrum of the reduction intermediate shows the absorption at 3371, 1637, 1270, and 1151 cm^−1^ for the OH, C = O, C-O and P = N stretching, respectively.

**Figure 1:**
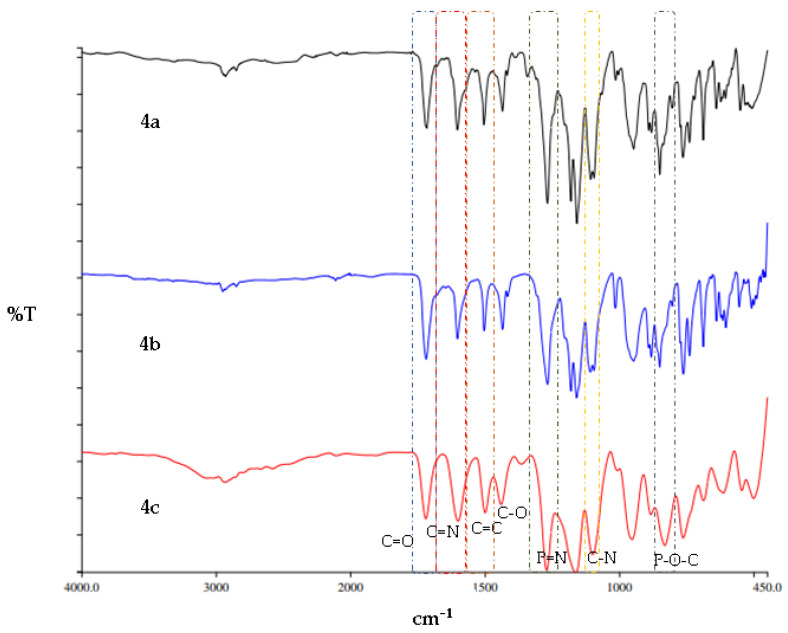
The overlay FTIR spectra of compounds **4a**–**c**.

The FTIR spectra of hexasubstituted cyclotriphosphazene compounds containing the Schiff base and ester linking unit attached to the electron withdrawing terminal group (Figure 1) showed the absorption band for C = N, C = C and C-O stretching at 1617 cm^−1^, 1535 cm^−1^ and 1238 cm^−1^, respectively. Absorption at 1182 cm^−1^ was assigned to P = N stretching, while the absorption at 1011 cm^−1^ was assigned for P-O-C bending. The absorption band for C-Cl bending of compound **4a** was observed at 842 cm^−1^.

### 3.3. NMR Spectral Discussion

**Figure 2:**
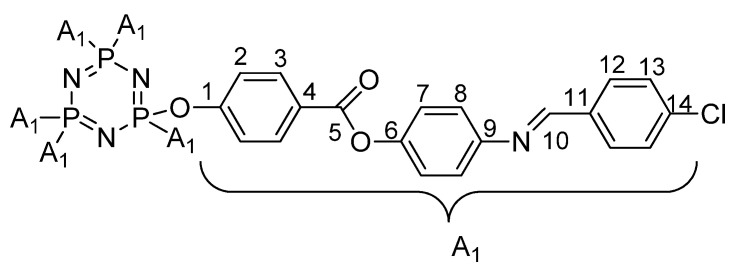
Chemical structure of compound **4a** with complete atomic numbering.

Structure confirmation of this series used compound **4a** as the representative. The structure of compound **4a** with complete atomic numbering is shown in Figure 2.

**Figure 3:**
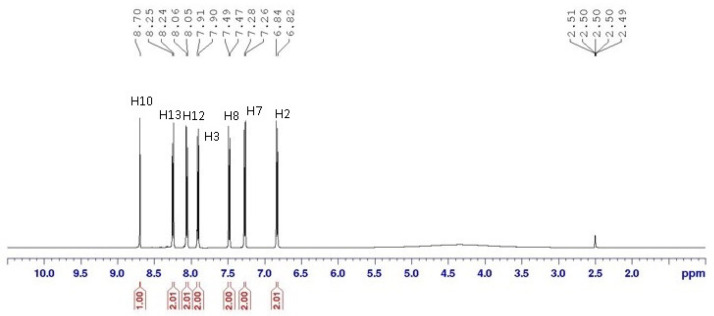
^1^H NMR (500 MHz, DMSO-d_6_) of compound **4a**.

The ^1^H NMR spectra of compound **4a** (Figure 3) showed a singlet of the Schiff Base proton, H10 was observed at the most downfield region (δ 8.70 ppm). Peaks at δ 8.25, 8.06, 7.91, 7.48, 7.27, and 6.83 ppm were assigned to the aromatic proton H13, H12, H18, H7, and H3, respectively. H13 experiences a greater deshielding effect compared to H2 because the chlorine atom decreases the electron density of molecules.

**Figure 4:**
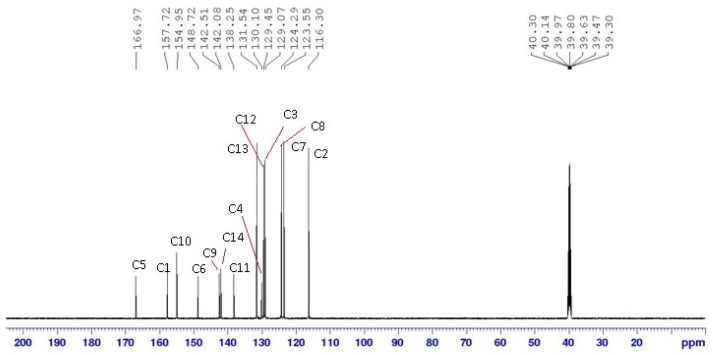
^13^C NMR (125 MHz, DMSO-d_6_) of compound **4a**.

According to the ^13^C NMR spectrum (Figure 4), compound **4a** showed one Schiff base, seven quaternary and six aromatic carbons signal. The signal of the Schiff base carbon, C15 was seen at 154.95 ppm. The aromatic resonated at 157.72 (C1), 148.72 (C6), 142.51 (C9), 142.08 (C14), 138.25 (C11) and 130.10 (C4) ppm. The signal of carbonyl carbon (C5) was observed in the downfield region of 166.97 ppm, which was followed by C1 as the carbon directly attached to the oxygen atoms. C6 was deshielded compared to C9 due to the electronegativity of an oxygen atom being higher than nitrogen atoms, which leads to a lower electron density around carbon; in accordance, greater deshielding on the former carbon could be observed.

**Figure 5:**
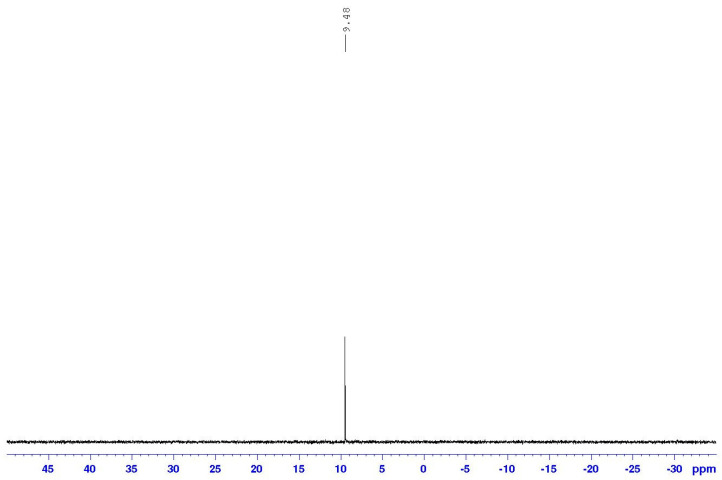
^31^P NMR (500 MHz, DMSO-d_6_) of compound **4a**.

The ^31^P NMR spectrum for compound **4a** (Figure 5) showed a singlet at 9.48 ppm, which indicates that all phosphorus was substituted identically in the side arms.

### 3.4. Thermogravimetric Analysis (TGA)

**Figure 6:**
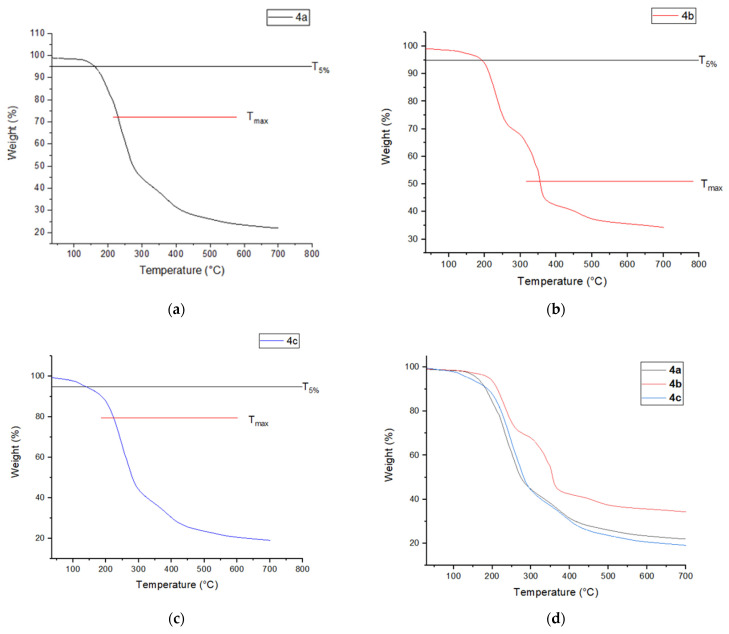
TGA curves for (**a**) compound **4a**, (**b**) compound **4b**, (**c**) compound **4c** and (**d**) all the final compounds containing electron withdrawing group as terminal end from 50 to 700 °C at a heating rate of 10 °C/min under N_2_ flow of 30 mL/min.

Thermal stabilities of the final compound were assessed by thermogravimetric analysis (TGA) under nitrogen atmosphere. Figure 6 shows the TGA data that has been plotted for weight (%) versus temperature from 0 to 700 °C. Based on the data shown in Table 2, it is revealed that no weight loss was detected at 96 °C for all compounds. Since all the final compounds contains a P-N skeleton, it will lead to excellent thermal properties since the phosphorus atom can act in the condensed phase promoting char formation, and the nitrogen atom will help in decomposing nitrogenous nonflammable gasses and will dilute oxygen concentration at the substrate–flame interface; hence, will develop the possibility of self-extinguishment [38]. At 5% weight loss, the final compound with the nitro group as the terminal end shows the highest result which is 192.84 °C compared to other compounds containing chloro and hydroxy group, which occurred at 141.01 °C and 169.69 °C, respectively. At the most rapid temperature, the weight loss of the final compound containing nitro group as terminal end remain higher, which is 189.73 °C compared to the other final compound containing chloro and hydroxy group at terminal end, which are 162.13 and 182.21 °C, respectively. The char residue for all final compound also has been observed to indicate which compound have an excellent thermal stability. The char residue at 700 °C showed that the compound with NO_2_ group at the terminal end has the highest char residue which is 34.2%. This can indicate that these compounds have great thermal stabilities due to a high formation of char yield. The creation of char may reduce the amount of combustible gas emitted, lessen the exothermicity brought on by the pyrolysis reaction, reduce the conductivity of the surfaces of burning materials resulted the ignition of the materials [39,40,41]. Based on this result, this compound also has promising properties and is effective in protecting the material at high temperatures and reducing the decomposition of the polymer.

### 3.5. Fire Retardant Testing

In this research, fire-retardant properties have been studied to observe the enhancement of epoxy resin towards being fire retardant when incorporated with a hexasubstituted cyclotriphosphazene compound containing an electron-withdrawing side arm. Only 1 wt.% was used in order to achieve less addictive usage. One wt.% of the final compound and mixed with epoxy resin and the value of LOI (FTT oxygen index, according to BS2782 Part 1: Method 141 and ISO 4589) was expressed in percentage and calculated according to the formula stated below:

LOI (%) = CF + kd(1)

where CF: the oxygen concentration of the final test, k: is the factor obtained from the manual book Fire Testing Technology, and d: is the oxygen concentration increment.

The compounds that exhibited an LOI value of 25% and above are considered self-extinguished [42], which means the compound has an ability to refrain from burning where the source of a flame has been. Table 3 shows the LOI value of each compound tested in this study.

According to the LOI data in Table 3, pure epoxy resin obtained an LOI value of 22.75%. The value of LOI for epoxy resin increased to 24.71% when incorporated with HCCP. This indicates that the presence of phosphorus (P) and nitrogen (N) atoms in the cyclotriphosphazene ring increases material resistance towards ignition. Another comparison can be drawn between a compound containing HCCP and the final compounds containing the electron-withdrawing side arm with Schiff base and ester linking units. The LOI data showed that the final compounds have higher LOI values than HCCP. This phenomenon was attributed to the Schiff base molecules promoting the formation of char on the condensed phase surfaces, which led to fire retardation [43]. Contact with combustion and fire spreading will be interrupted and prevented by removing heat from materials that can burn and also by the creation of char during fire. Moreover, the star-shaped structure of HCCP increases the aromatic ring concentration, which is helpful for the formation of coherent char layers and preventing the emergence of combustible carbon-containing volatile [44]. Besides that, among these three electron-withdrawing side arms, the NO_2_ and Cl side arms obtained high LOI values of 26.71% and 26.90%, respectively. This observation might be supported by evidence that NO_2_ and Cl electron-withdrawing properties improve the P–N bond synergistic effect [45]. This also leads to this compound exhibiting condensed and gas phases that protect it from burning. Thus, this compound possesses a high LOI value. The compound with a hydroxyl group substituent at the side arm shows an LOI value of 26.53%. The presence of the small group, which corresponds to the lone pair of an electron, improves the resin’s ability to adhere. Inter-hydrogen bonds are simply an attraction between partially positive hydrogen and partially negative or negative atoms created by the electron in the terminal group. The presence of hydrogen bonds increased the compound’s thermal stability and fire resistance, resulting in a significantly higher LOI value [46,47].

### 3.6. Residue Analysis

**Figure 7:**
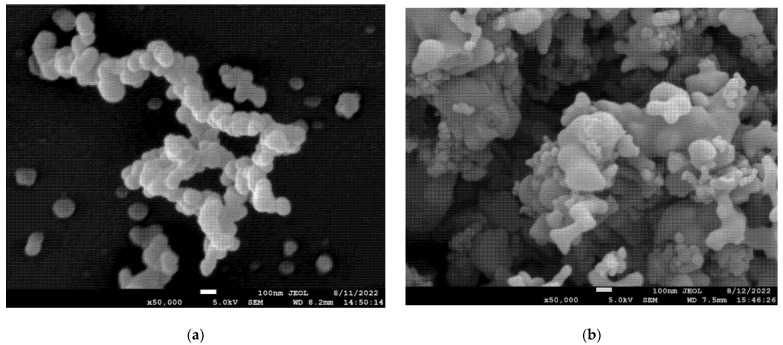
SEM images of char residues of (**a**) pure epoxy resin and (**b**) epoxy resin + 1 wt.% of compound **4b**.

The char residue analysis of the final compound incorporated with epoxy resin after the LOI test characterized by SEM is shown in Figure 7 in order to observe the microscale morphology of the studied materials. The residue of epoxy resin and compound **4b** was used as a representative of other compounds as this compound showed a high LOI value. Based on the SEM image (Figure 7b), the char layer epoxy resin incorporated with the final compound containing the nitro group at the terminal end is dense and agglomerated which indicate that the compound efficiently prevents flammable gas from escaping inside. It also demonstrates how this substance can create a layer of char that is more stable and has great thermal oxidation resistance, which prevents the release of combustible gas products or free radicals and significantly improves the self-extinguishing properties [13], while the presence of single circular chains (Figure 7a) on the char surface of pure epoxy resin can promote the rapid transfer of heat and combustible gases that accelerates the burning. This result was in alignment with the LOI value obtained for pure epoxy resin that show the lowest value of LOI and thus indicate that the epoxy resin has a poor fire performance ability.

### 3.7. Dielectric Properties Testing

Lastly, this research aimed to investigate the dielectric properties, which is the dielectric strength of epoxy resin incorporated with the final compounds that contain the Schiff base and ester linking unit with an electron-withdrawing as side arm. The dielectric properties are measured by Alternating current breakdown voltage. The sample was prepared according to the standard method ASTM D149, the thickness of the sample used was 2 mm. The value of the breakdown voltage was expressed in kV (kilovolt), and the formula used to calculate the breakdown voltage is as follows:

Dielectric Strength (kV/mm^−1^) = Breakdown voltage (kV)/Thickness of sample (mm).

**Figure 8:**
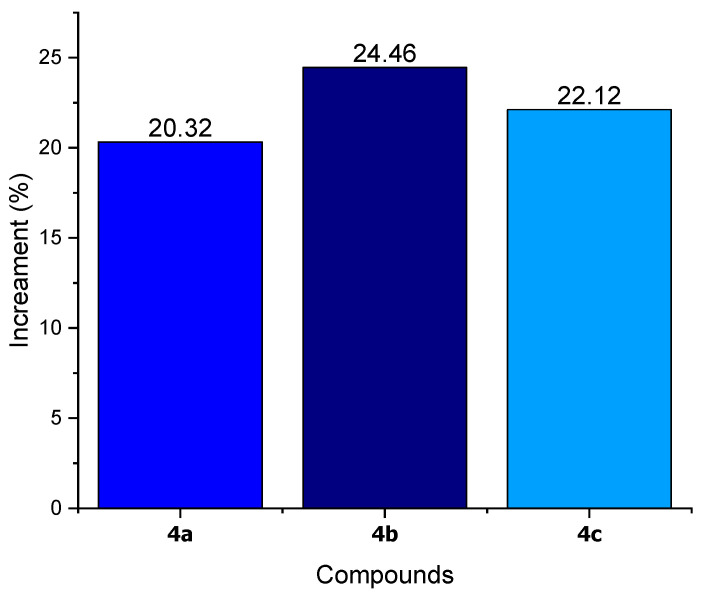
Increment of dielectric breakdown strength of compounds **4a**–**c**.

Table 4 revealed the breakdown strength of pure epoxy resin and the final compound with the electron-withdrawing side arm, which are Cl, NO_2_, and OH at the terminal end incorporated with epoxy resin. Based on Table 4, the breakdown voltage of the pure epoxy resin was 19.54 kV/mm^−1^. While the dielectric strength for the final compounds with Cl, NO_2_, and OH at the terminal end was 23.51, 24.31, and 23.86 kV/mm^−1^, respectively. The breakdown voltage of epoxy resin incorporated with the final compound obtained was higher compared to the pure epoxy resin. Among the final compound that contain electron withdrawing group as terminal end, compounds with nitro at the terminal end have a higher breakdown voltage. This is due to the effect of the nitro group, especially at the para position with respect to the electron-donating functional group is well known to cause electron displacement π electron delocalization [48] that leads to high dielectric strength. In addition, the dielectric strength obtained from the compound containing a chlorine atom in the side arm is 23.51 kV/mm^−1^. The final compound containing a chlorine atom in the side arm also shows the promising result on dielectric strength due to its high electronegativity [49]. Thus, this compound might have the highest potential to be used to form electrical applications because it shows an outstanding result of dielectric properties, while for Figure 8, the percentage of increment has been calculated to shows the enhancement of breakdown voltage performance between pure epoxy resin and epoxy resin incorporated with the final compound. The highest percentage value of increment drawn by the epoxy resin incorporated with final compound containing a nitro group at the terminal end is 24.41%; it also revealed the highest breakdown voltage among all of the final compound.

## 4. Conclusions

A series of hexasubstituted cyclotriphosphazene compounds with Schiff base and ester linking units were successfully synthesized and characterized. These homologues were differentiated with chloro, nitro and hydoxy terminal groups. The final compound was then incorporated with epoxy resin to determine the compounds’ fire retardancy and dielectric properties. The LOI test showed that the final compounds with nitro group (NO_2_) terminal end showed the highest LOI value of 26.90%. TGA analysis showed the final compound containing the nitro group has the highest weight loss at 189.73 °C and char residue, which is 34.2%. The presence of a nitro group able to induce the fire retardation by enhancing the synergistic effect of P–N bonds. The thermal stabilities of the epoxy resin incorporated with the final compound were better than the pure epoxy resin, which indicate that the final compound provide a positive influence in the flame retardance of epoxy resin. While for dielectric properties testing, the same compound also showed the highest breakdown voltage, which is 24.31 kV/mm^−1^ due to the π electron delocalization of the nitro group. Moreover, this compound may be further used as an addictive in epoxy resin to restrain the limitation of epoxy resin in high fire performance requirements with high voltage compound application.

## Figures and Tables

**Table 1:** CHN elemental analysis for compounds **4a**–**c**.

Element	Compound 4a		Compound 4b		Compound 4c	
	Theoretical (%)	Experimental (%)	Theoretical (%)	Experimental (%)	Theoretical (%)	Experimental (%)
C	64.36	64.27	62.59	62.50	67.70	67.53
H	3.51	3.48	3.41	3.35	3.98	3.88
N	5.63	5.58	9.12	9.06	5.92	5.87

**Table 2:** Thermal properties of final compounds, **4a**–**c**.

Compounds	Temperature of 5% Weight Loss (°C)	Temperature of most Rapid Weight Loss (°C)	Residue at 700 (%)
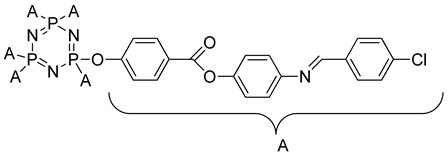 Compound **4a**	160.89	162.13	22.1
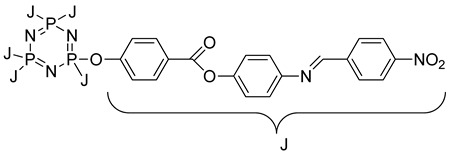 Compound **4b**	192.88	189.78	34.2
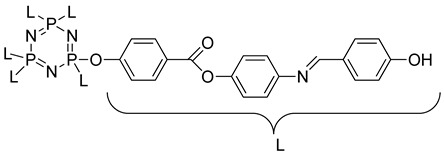 Compound **4c**	141.01	186.21	19.07

**Table 3:** LOI value of the compounds.

Compound	Limiting Oxygen Index (%)	Side Arm
Pure Epoxy Resin	22.75 (±0.00)	-
Epoxy Resin + HCCP	24.71 (±0.00)	-
Epoxy Resin + 1 wt.% Compound **4a**	26.71 (±0.00)	–Cl
Epoxy Resin + 1 wt.% Compound **4b**	26.90 (±0.00)	–NO_2_
Epoxy Resin + 1 wt.% Compound **4c**	26.53 (±0.00)	–OH

**Table 4:** Breakdown voltage of the compounds.

Compound	Breakdown Voltage (kV/mm^−1^)	Side Arm
Pure Epoxy Resin	19.54	-
Epoxy Resin + 1 wt.% Compound **4a**	23.51	–Cl
Epoxy Resin + 1 wt.% Compound **4b**	24.31	−NO_2_
Epoxy Resin + 1 wt.% Compound **4c**	23.56	−OH

## Data Availability

Not applicable.
